# Differences in complication patterns in subgroups of type 2 diabetes according to insulin resistance and beta-cell function

**DOI:** 10.1038/s41598-022-13084-6

**Published:** 2022-06-07

**Authors:** Yongin Cho, Seong Ha Seo, Da Hea Seo, Seong Hee Ahn, Seongbin Hong, Byung Wook Huh, Yong-ho Lee, Seok Won Park, Young Ju Suh, So Hun Kim

**Affiliations:** 1 Division of Endocrinology and Metabolism, Department of Internal Medicine, Inha University College of Medicine, Incheon, Republic of Korea; 2 Huh’s Diabetes Center and the 21st Century Diabetes and Vascular Research Institute, Seoul, Republic of Korea; 3 Department of Internal Medicine, Yonsei University College of Medicine, Seoul, Republic of Korea; 4 Department of Biomedical Sciences, Inha University College of Medicine, Incheon, Republic of Korea

**Keywords:** Endocrinology, Medical research

## Abstract

This study aimed to determine whether the patterns of diabetic complications differed when patients with type 2 diabetes mellitus (T2DM) were simply classified according to insulin sensitivity and beta-cell function. This observational study included 8861 patients with T2DM who underwent concurrent testing for fasting glucose, fasting insulin, and one or more diabetic complications. We categorized the patients into four groups according to insulin sensitivity and beta-cell function. Compared with the reference group (mild insulin resistance and beta-cell dysfunction), the “severe beta-cell dysfunction” group had lower odds of chronic kidney disease [adjusted odds ratios (aOR) 0.611]. The “severe insulin resistance” group had higher odds of carotid artery plaque presence (aOR 1.238). The “severe insulin resistance and beta-cell dysfunction” group had significantly higher odds of large fiber neuropathy (aOR 1.397, all *p* < 0.05). After a median of five years of follow-up, this group distinction did not lead to a difference in risk of new diabetic retinopathy or chronic kidney disease. In addition, there was no significant difference among the groups in plaque progression risk over 8–10 years in the longitudinal follow-up analysis. The patterns of complications differ when patients with T2DM are classified according to insulin resistance and beta-cell dysfunction. However, there were no differences in the risk of developing new complications.

## Introduction

Type 2 diabetes mellitus (T2DM) is characterized by two major metabolic abnormalities: insulin resistance and insulin secretory dysfunction^1^. However, the contribution of each factor to the development and progression of T2DM varies among individuals. T2DM is highly heterogeneous with regard to clinical presentation and progression of complications. Efforts have been made to classify T2DM into several subtypes on the basis of the distinct patterns of clinical characteristics and disease comorbidities^2^. Ahlqvist et al.^3^ classified patients with newly diagnosed diabetes by using a clustering technique based on six variables: anti-glutamate decarboxylase antibodies (GADAs), age at diagnosis, body mass index (BMI), HbA1c levels, and homeostasis model assessment (HOMA) 2 estimates of beta-cell function and insulin resistance. They confirmed that differences exist in disease progression and the risk of diabetic complications between the groups. A recent study involving Japanese patients reported similar findings^4^.

Classifying patients using the abovementioned clustering technique has many advantages, but this method may be difficult to apply during patient treatment in some cases. It is often difficult to perform a full evaluation, including testing for GADAs, in patients with new-onset diabetes. The clustering technique was performed for patients with newly diagnosed diabetes (within one year). However, the variables used for clustering were not necessarily measured at the onset of diabetes. Another limitation is that it is difficult to classify patients who have received diabetes treatment for a long duration by using the clustering analysis described above. Given that T2DM is rarely diagnosed immediately upon onset, the duration of exposure to hyperglycemia in patients with diabetes may vary^5^.

In this study, we attempted to investigate the clinical significance of classifying patients with T2DM on the basis of only insulin resistance and beta-cell dysfunction, which are two of the major pathologies of T2DM, and determined whether there is a difference in complication risk between groups. Furthermore, we attempted to confirm whether this classification method is associated with future complication risk.

## Subjects, materials and methods

### Study population

This study was an observational retrospective study that used data from 13,296 patients with T2DM enrolled in the Seoul Metabolic Syndrome Cohort from November 1997 to September 2016 at Huh’s Diabetes Center^6–9^. All patients with T2DM were recommended to conduct a regular complication study in accordance with the guidelines of the Korean Diabetes Association. Patients who consented to the test were screened for diabetes complications. We analyzed the data of patients who underwent concurrent testing for fasting glucose, fasting insulin, and one or more complications within six months from the time of enrollment. Patients with clinically suspected type 1 diabetes or latent autoimmune diabetes in adults with positive result of GADAs were excluded. Considering the possible effect of the remaining exogenous insulin in the body, patients using insulin at baseline were also excluded.

A total of 8861 patients with T2DM were enrolled in the current study. The diagnosis and classification of T2DM was based on the classification of the American Diabetes Association. All participants provided written informed consent, and the ethics committee of Inha University Hospital approved this study (2021-04-033).

### Definitions of diabetes subgroups

Participant information, such as medical and family history, smoking and alcohol history, physical activity level per week, and medication history were collected. Anthropometric data including weight, height, and waist circumference (WC) were obtained, and blood samples were collected by trained nurses from participants after ≥ 8 h of fasting. BMI was calculated as weight divided by height squared (kg/m^2^). The estimated glomerular filtration rate (eGFR) was calculated using the formula of the Chronic Kidney Disease Epidemiology Collaboration.

Insulin resistance was assessed using the HOMA for insulin resistance (HOMA-IR) with the following formula: fasting insulin (μU/mL) × fasting glucose (mg/dL)/405^10^. Pancreatic beta-cell function was assessed using the HOMA for beta-cell function (HOMA-B) with the following formula: 360 × fasting insulin (μU/mL)/(fasting glucose [mg/dL]—63)^11^. High insulin resistance was defined as a HOMA-IR value higher than the median value for the respective sex, and beta-cell dysfunction was defined as having a HOMA-B value lower than the median value for the respective sex. The patient group without insulin resistance above median or beta-cell dysfunction was classified as group 1 (mild insulin resistance and beta-cell dysfunction group, reference group). Patients with severe beta-cell dysfunction and mild insulin resistance, severe insulin resistance and mild beta-cell dysfunction, and both severe insulin resistance and beta-cell dysfunction were classified into groups 2, 3, and 4, respectively. Blood samples were collected in the fasting state without the use of insulin. Insulin was measured using chemiluminescence immunoassay Centaur® XP (Siemens Healthcare) until February 20, 2015. From February 21, 2015, insulin was measured using a Roche Cobas e801 (Roche Diagnostics) electrochemiluminescence immunoassay. Differences in test methods can affect group classification and result values, so the differences in the methods were defined as additional correction variables.

### Definitions of diabetic complications

Diabetic retinopathy was defined as the presence of 1 or more retinal microaneurysms or retinal blot hemorrhages on the fundus photographs with or without more severe lesions including exudates, venous bleeding, new retinal vessels, and fibroproliferations^12^. The presence of hepatic steatosis was assessed using abdominal ultrasonography (iU22; Philips Healthcare, Andover, MA, USA) with a 3.5 MHz transducer after 8 h of fasting^13^. As previously described, the degree of steatosis was assessed semi-quantitatively (absent, mild, moderate, or severe)^14^. Large fiber diabetic polyneuropathy was identified among those who met the respective diagnostic criteria on the basis of the results of nerve conduction velocity tests^15^. Chronic kidney disease (CKD) was defined as an eGFR < 60 mL/min/1.73 m^2^. The common carotid arteries were examined using high-resolution ultrasonography (LOGIQ7; GE Healthcare, Chicago, IL, USA). The presence of carotid plaque was confirmed if any one of the following criteria was met: (1) carotid intima-media thickness of 1.5 mm or higher, (2) protrusion of atherosclerosis into the lumen of the artery with ≥ 50% thickness compared to the surrounding area, and (3) presence of distinct areas of hyperechogenicity^9,16^. All ultrasound examinations were performed by trained radiologists who were blinded to the patients’ clinical and laboratory information.

To analyze the progression of carotid atherosclerosis, the results of repeated carotid artery ultrasonography after 8–10 years of follow-up were also collected. In this analysis, patients with bilateral carotid artery plaques at baseline in whom the occurrence of new-onset plaque was difficult to judge on repeat ultrasonography were excluded. The progression of carotid atherosclerosis was defined as the appearance of newly developed carotid plaque lesions on repeat ultrasonography. The risk of newly developed retinopathy and renal insufficiency was also evaluated on the basis of fundoscopy and blood sample examination, which were followed up regularly. For retinopathy, the risk of newly occurring retinopathy was evaluated in patients with two or more annual examinations including baseline examination. In patients with three or more annual eGFR examinations, new CKD development was defined when an eGFR of less than 60 mL/min/1.73 m^2^ was confirmed twice or more consecutively. Analysis of newly developed polyneuropathy could not be performed due to the small number of patients who underwent follow-up nerve conduction velocity tests.

### Statistical analysis

The baseline characteristics of the study participants were analyzed according to the four groups. Data are presented as mean ± standard deviation for normally distributed continuous variables, median with interquartile range (IQR) for non-normally distributed continuous variables, or as numbers (percentages) for categorical variables. Normally distributed continuous variables were analyzed using one-way analysis of variance for inter-group comparisons, followed by the Bonferroni test for post hoc analysis. Non-normally distributed continuous variables were analyzed using Kruskal–Wallis test for inter-group comparisons, followed by the Dunn procedure for post hoc analysis. All categorical variables were expressed as numbers (proportions) and were compared using the χ^2^ test.

Multiple logistic regression analysis was performed to evaluate the statistical significance of the risk of accompanying diabetic complications in the study groups. The results were adjusted for age, sex, diabetes duration, systolic blood pressure, diastolic blood pressure, method of insulin measurement, time at enrollment, BMI, HbA1c levels, LDL cholesterol levels, eGFR (not included in the analysis of CKD risk), smoking status, alcohol consumption, and physical exercise. Adjusted odds ratios (aORs) and 95% confidence intervals (CIs) were determined.

Cox regression was also performed to evaluate the statistical significance of the risk of new onset diabetic complications in the study groups. Events were counted from baseline examination to the 1^st^ newly developed event (as defined each), and patients without an event had their data censored at the date of last examination. The results were adjusted for the same variables without method of insulin measurement (which affects greatly on the observation period) as multiple logistic regression analysis. Adjusted hazard ratios and 95% CIs were also determined for new developments of diabetic retinopathy, CKD, and carotid plaque that had regular examination and follow-up results. Statistical analyses were performed using IBM SPSS statistical software for Windows (version 26.0; IBM, Armonk, NY, USA). Statistical significance was set at *p* < 0.05.

## Results

Among the 8861 T2DM patients, 4226 patients (47.7%) were women. The average age was 57.4 years, the median diabetes duration was 5.0 years, and the average BMI was 24.5 kg/m^2^. Abnormalities in insulin sensitivity and beta-cell function values were defined on the basis of the median value for each sex (male: 2.44 for HOMA-IR and 32.1 for HOMA-B; female: 2.47 for HOMA-IR and 39.1 for HOMA-B). Patients with T2DM were classified into four groups according to insulin sensitivity and beta-cell function. Group 1, which included 2044 patients (23.1%) who had mild insulin resistance (HOMA-IR under median) and had preserved beta-cell function (HOMA-B above median), was characterized by a short T2DM duration and late-onset disease; this group was labeled as the mild insulin resistance and beta-cell dysfunction (reference) group. Group 2, which included 2382 (26.9%) patients who had mild insulin resistance and had severe beta-cell dysfunction (labeled as the severe beta-cell dysfunction group), was characterized by long T2DM duration, low BMI, and high HDL cholesterol. Compared with group 1, group 2 had significantly higher HbA1c and LDL cholesterol levels. Group 3, which included 2390 (27.0%) patients who were more insulin resistant and had mild beta-cell dysfunction (labeled as the severe insulin resistance group), was characterized by high WC, low HDL cholesterol, and high BMI. The prevalence of CKD, hepatic steatosis, and carotid plaque was the highest in this group. Group 4, which included 2045 (23.1%) patients who had severe insulin resistance and severe beta-cell dysfunction (labeled as the severe insulin resistance and beta-cell dysfunction group), was characterized by a long T2DM duration, early onset disease, high HbA1c, and high LDL cholesterol. The prevalence of retinopathy and large fiber neuropathy was the highest in this group (Table 1).

**Table 1:** Baseline clinical characteristics in the patient groups classified according to insulin sensitivity and beta-cell function.

Total n = 8861	Group 1 Mild insulin resistance and beta-cell dysfunction (Reference)	Group 2 Severe beta-cell dysfunction	Group 3 Severe insulin resistance	Group 4 Severe insulin resistance and beta-cell dysfunction	*p* value
	n = 2044	n = 2382	n = 2390	n = 2045	
Female, n (%)	968 (47.4)	1144 (48.0)	1145 (47.9)	969 (47.4)	0.956
Age (years)	58.3 ± 10.7	58.1 ± 10.2	57.8 ± 11.1	55.2 ± 11.3*^†‡^	** < 0.001**
DM duration (years)	3.0 (9.0)	7.0 (10.0)*	4.0 (9.0)*^†^	7.0 (9.0)*^‡^	** < 0.001**
Age at DM onset (years)	53.2 ± 10.5	49.8 ± 10.3*	51.9 ± 10.7*^†^	46.6 ± 10.4*^†‡^	** < 0.001**
Diabetes medications					** < 0.001**^Ω^
Without medication	770 (37.7)	699 (29.3)	687 (28.7)	634 (31.0)	
One OAD only	624 (30.5)	564 (23.7)	737 (30.8)	399 (19.5)	
Two OADs	534 (26.1)	817 (34.3)	742 (31.0)	673 (32.9)	
Three OADs	109 (5.3)	288 (12.1)	218 (9.1)	323 (15.8)	
Four or more OADs	7 (0.3)	14 (0.6)	6 (0.3)	16 (0.8)	
SBP (mmHg)	132.1 (17.1)	132.7 (17.5)	136.0 (17.7)*^†^	133.9 (18.2)*^‡^	** < 0.001**
DBP (mmHg)	84.2 (11.1)	84.1 (11.1)	86.4 (11.5)*^†^	85.4 (11.5)*^†‡^	** < 0.001**
Body weight (kg)	64.2 ± 18.9	62.6 ± 10.4*	69.0 ± 12.0*^†^	65.3 ± 11.6^†‡^	** < 0.001**
BMI (kg/m^2^)	24.1 ± 3.1	23.6 ± 2.9*	25.9 ± 3.4*^†^	24.3 ± 3.1^†‡^	** < 0.001**
WC (cm)	82.3 ± 8.6	81.6 ± 8.4*	87.2 ± 8.6*^†^	83.9 ± 8.8*^†‡^	** < 0.001**
HOMA-IR	1.8 (0.6)	1.6 (0.9)*	3.6 (1.8)*^†^	3.5 (1.5)*^†^	** < 0.001**
HOMA-B	53.0 (26.4)	20.9 (15.0)*	62.0 (43.3)*^†^	20.2 (13.3)*^‡^	** < 0.001**
HbA1c (%)	6.9 ± 1.1	8.2 ± 1.7*	7.7 ± 1.3*^†^	9.9 ± 1.9*^†‡^	** < 0.001**
Total cholesterol (mg/dL)	188.2 ± 38.0	195.1 ± 40.7*	193.9 ± 39.1*	205.1 ± 45.2*^†‡^	** < 0.001**
HDL cholesterol (mg/dL)	50.5 ± 15.1	52.2 ± 14.0*	47.5 ± 11.9*^†^	49.7 ± 12.6^†‡^	** < 0.001**
Triglyceride (mg/dL)	130.9 ± 89.8	131.4 ± 92.3	165.0 ± 115.6*^†^	175.3 ± 147.9*^†^	** < 0.001**
LDL cholesterol (mg/dL)	111.9 ± 33.5	117.2 ± 36.4*	113.7 ± 34.5^†^	121.6 ± 38.7*^†‡^	** < 0.001**
eGFR (EPI)	87.8 ± 18.6	87.9 ± 18.4	86.5 ± 19.3	89.6 ± 20.0*^†‡^	** < 0.001**
AST (IU/L)	26.1 ± 10.4	28.4 ± 17.5*	30.1 ± 18.3*^†^	27.3 ± 20.4^‡^	** < 0.001**
ALT (IU/L)	26.4 ± 17.4	27.5 ± 19.5	34.6 ± 25.9*^†^	31.1 ± 29.5*^†‡^	** < 0.001**
Use of statin	270/2044 (13.2)	339/2382 (14.2)	389/2390 (16.3)	283/2045 (13.8)	**0.021**^**Ω**^
History of					
History of Hypertension	565/2044 (27.6)	642/2382 (27.0)	808/2390 (33.8)	527/2045 (25.8)	** < 0.001**^**Ω**^
Coronary heart disease	37/2044 (1.8)	48/2382 (2.0)	49/2390 (2.1)	17/2045 (0.8)	**0.006**^Ω^
Ischemic stroke	18/2044 (0.9)	21/2382 (0.9)	28/2390 (1.2)	17/2045 (0.8)	0.619
Peripheral artery disease	8/2044 (0.4)	7/2382 (0.3)	10/2390 (0.4)	2/2045 (0.1)	**0.220**
Diabetic retinopathy, n (%)	77/1799 (4.3)	161/2224 (7.2)	112/2226 (5.0)	185/1958 (9.4)	** < 0.001**^Ω^
Chronic kidney disease, n (%)	144/2002 (7.2)	143/2335 (6.1)	201/2348 (8.6)	171/2014 (8.5)	**0.004**^Ω^
Hepatic steatosis, n (%)	724/1619 (44.7)	839/1946 (43.1)	1391/1997 (69.7)	1.037/1722 (60.2)	** < 0.001**^Ω^
Large fiaber neuropathy, n (%)	228/1372 (16.6)	481/1929 (24.9)	377/1835 (20.5)	580/1706 (34.0)	** < 0.001**^Ω^
Carotid plaque, n (%)	814/1.762 (46.2)	1005/2035 (49.4)	1116/2147 (52.0)	952/1877 (50.7)	**0.003**^Ω^

Data are presented as mean ± standard deviation for normally distributed continuous variables, median with interquartile range (IQR) for non-normally distributed continuous variables, and number (%) for categorical variables.

*DM* diabetes mellitus, *SBP* systolic blood pressure, *DBP* diastolic blood pressure, *BMI* body mass index, *WC* waist circumference, *HOMA* homeostatic model assessment, *IR* insulin resistance, *HDL* high-density lipoprotein, *LDL* low-density lipoprotein, *eGFR* estimated glomerular filtration rate, *EPI* Epidemiology Collaboration, *AST* aspartate aminotransferase, *ALT* alanine aminotransferase.

Bold text indicates *p* values < 0.05.

*p values < 0.05, vs. group 1, by post hoc analyses (Bonferroni tests or Dunn procedure).

^†^*p* values < 0.05, vs. group 2, by post hoc analyses (Bonferroni tests or Dunn procedure).

^‡^*p* values < 0.05, vs. group 3, by post hoc analyses (Bonferroni tests or Dunn procedure).

^Ω^Significant chi-square test, *p* < 0.05.

**Figure 1:**
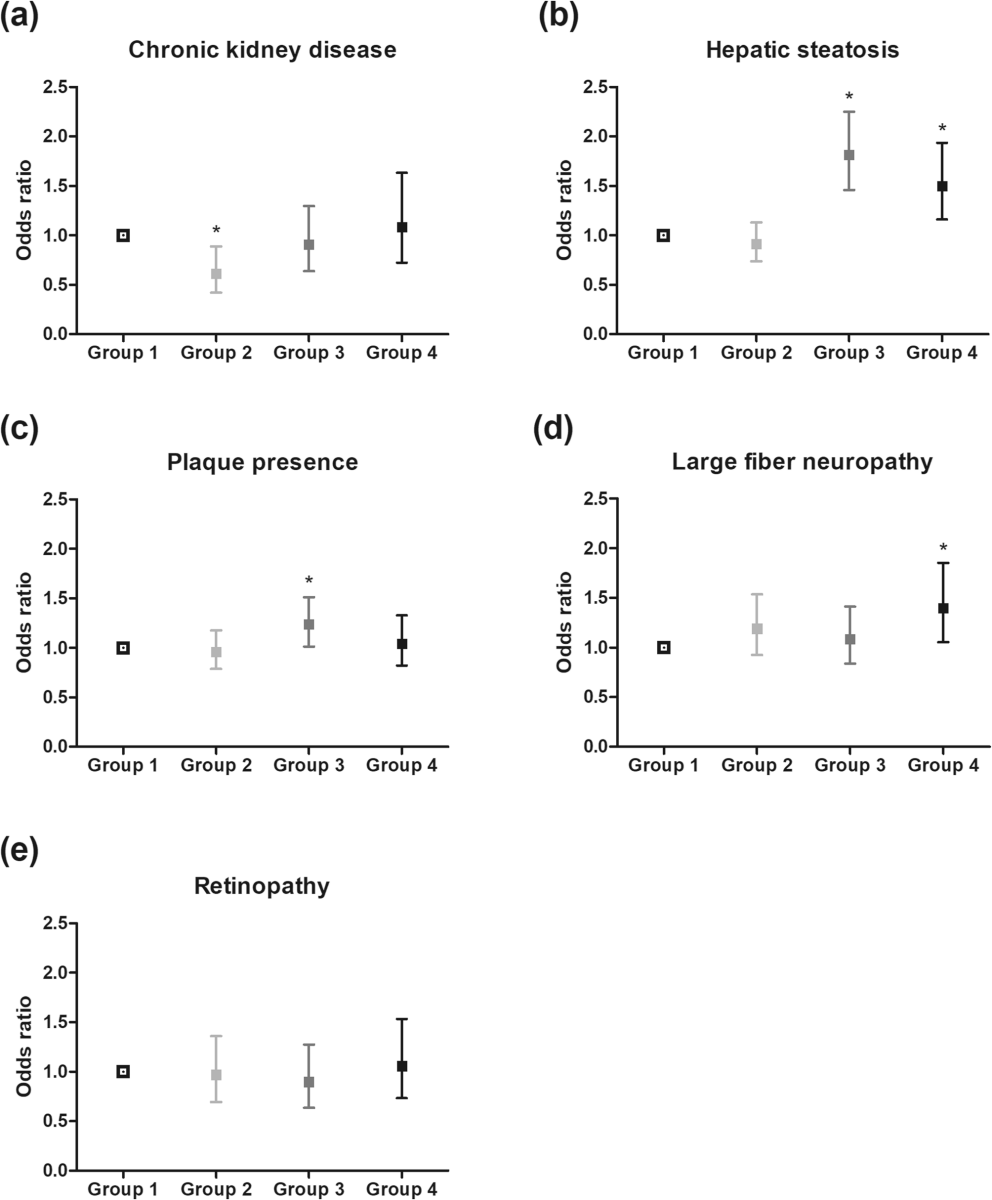
Odds of accompanying diabetic complications or hepatic steatosis by group according to insulin sensitivity and beta-cell function. Odds ratios of (**a**) chronic kidney disease (eGFR < 60 mL/min/1.73 m^2^), (**b**) hepatic steatosis, (**c**) plaque presence, (**d**) large fiber neuropathy, and (**e**) diabetic retinopathy. The results were adjusted for age, sex, diabetes duration, systolic blood pressure, diastolic blood pressure, time of enrollment, method of insulin measurement, BMI, HbA1c levels, LDL cholesterol levels, eGFR (not included in the analysis depicted in **a**), statin use, smoking status, alcohol consumption, and physical activity. Group 1. Reference (mild insulin resistance and beta-cell dysfunction) group. Group 2. “Severe beta-cell dysfunction” group. Group 3. “Severe insulin resistance” group. Group 4. “Severe insulin resistance and beta-cell dysfunction” group. *BMI* body mass index, *LDL* low-density lipoprotein, *eGFR* estimated glomerular filtration rate. *p < 0.05 versus group 1.

The odds of having diabetic complications were assessed by adjusting for age, sex, diabetes duration, systolic blood pressure, diastolic blood pressure, time of enrollment, method of insulin measurement, BMI, HbA1c levels, LDL cholesterol levels, eGFR (not included in the analysis of CKD risk), smoking status, alcohol consumption, and physical exercise. Compared with group 1 (mild insulin resistance and beta-cell dysfunction group), group 2 (severe beta-cell dysfunction group) had no higher prevalence of any diabetic complications. However, group 2 had lower odds of chronic kidney disease (eGFR < 60 mL/min/1.73 m^2^, aOR 0.611, 95% CI 0.420–0.889; Fig. 1a and Supplementary Table 1). Group 3 (severe insulin resistance group) had higher prevalence of hepatic steatosis (aOR 1.813, 95% CI 1.459–2.252; Fig. 1b) and carotid artery plaque presence (aOR 1.238, 95% CI 1.014–1.512; Fig. 1c). Group 4 (severe insulin resistance and beta-cell dysfunction group) had a significantly higher prevalence of hepatic steatosis (aOR 1.499, 95% CI 1.161–1.935; Fig. 1b) and large fiber neuropathy (aOR 1.397, 95% CI 1.055–1.851; Fig. 1d). The odds of diabetic retinopathy did not differ significantly between the groups (Fig. 1e). All of the above analyses were conducted once again after adjusting diabetic medications including sulfonylurea and thiazolidinedione which could have affected HOMA-IR and HOMA-B values. There was no meaningful difference in the final results (Supplementary Fig. 1).

**Figure 2:**
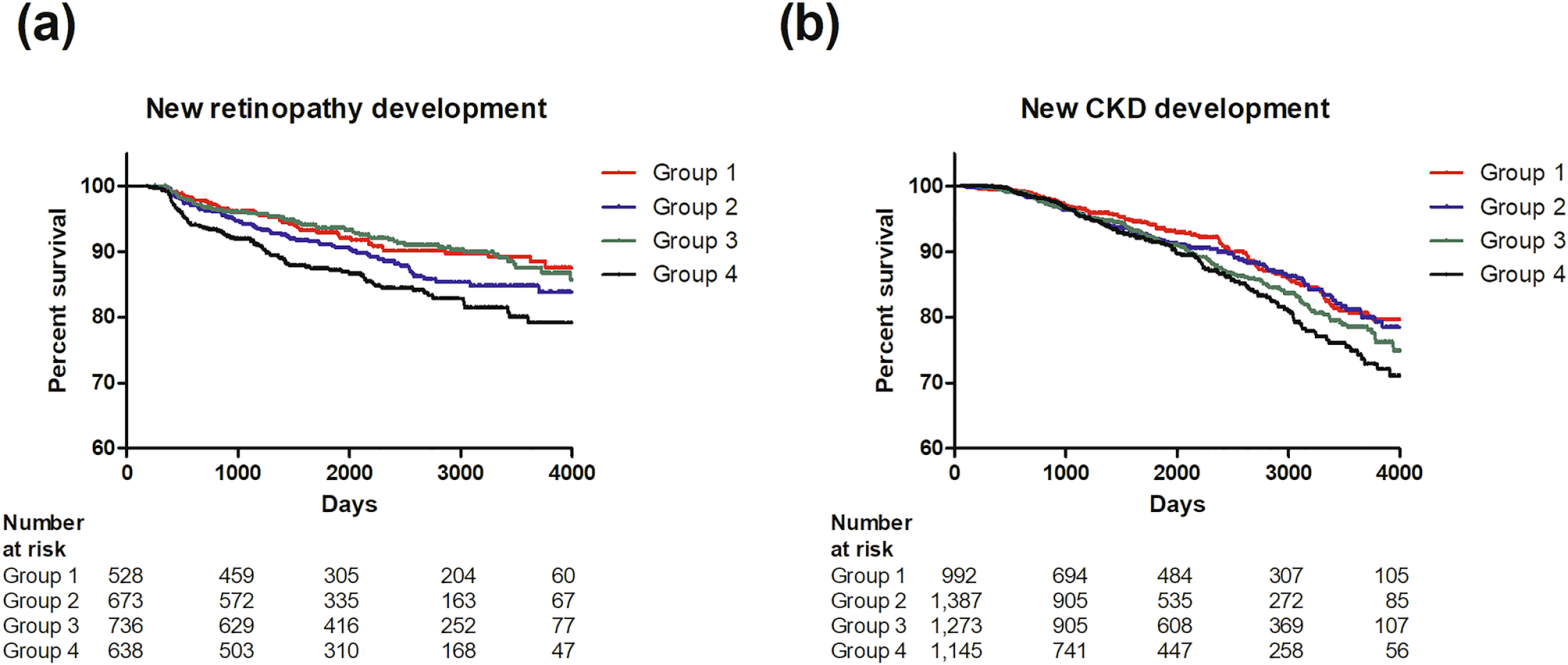
Risk of developing diabetic complications by group. Kaplan–Meier curve and hazard ratios of (**a**) retinopathy and (**b**) chronic kidney disease. The results were adjusted for age, sex, diabetes duration, systolic blood pressure, diastolic blood pressure, time of enrollment, BMI, HbA1c levels, LDL cholesterol levels, eGFR (not included in the analysis depicted by [**b**]), statin use, smoking status, alcohol consumption, and physical activity. Group 1. Reference (mild insulin resistance and beta-cell dysfunction) group. Group 2. “Severe beta-cell dysfunction” group. Group 3. “Severe insulin resistance” group. Group 4. “Severe insulin resistance and beta-cell dysfunction” group. *aHR* adjusted hazard ratio, *CKD* chronic kidney disease.

**Figure 3:**
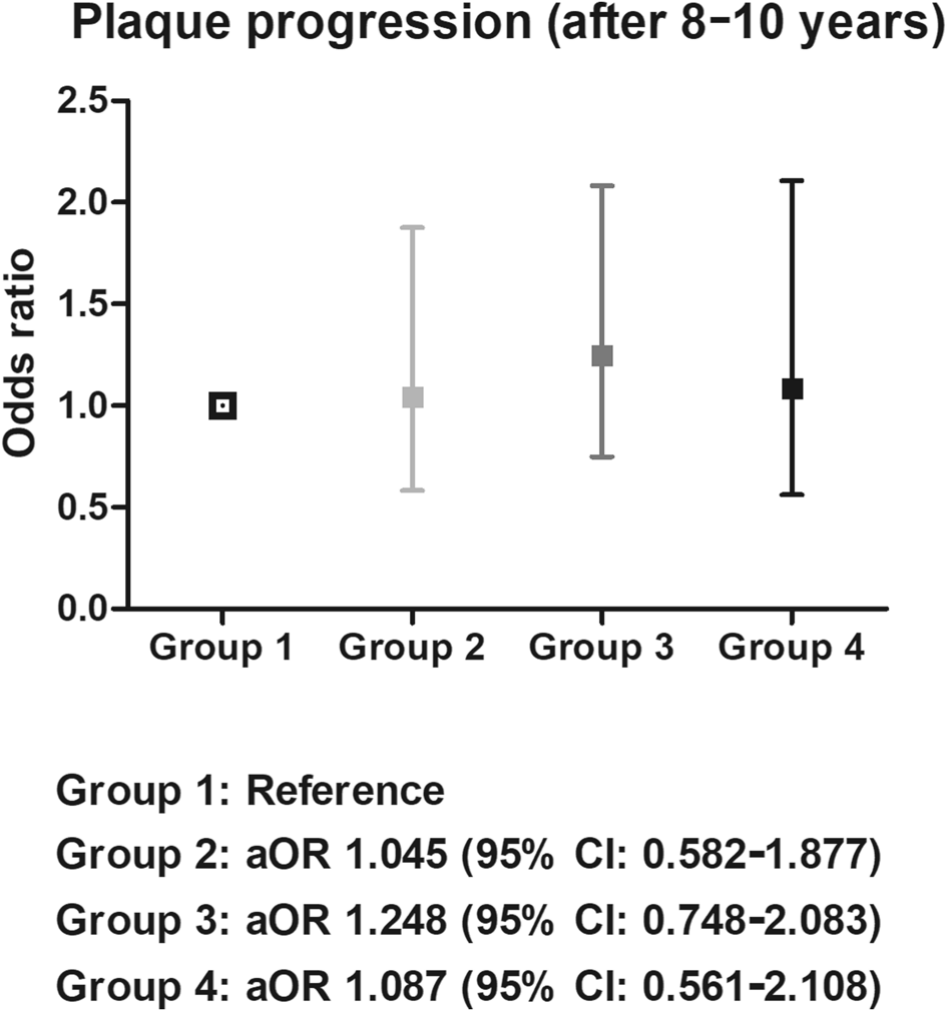
Risk of carotid plaque progression by group. Odds ratio of carotid plaque progression. The results were adjusted for age, sex, diabetes duration, systolic blood pressure, diastolic blood pressure, time of enrollment, BMI, HbA1c levels, LDL cholesterol levels, eGFR, statin use, smoking status, alcohol consumption, and physical activity. Group 1. Reference (mild insulin resistance and beta-cell dysfunction) group. Group 2. “Severe beta-cell dysfunction” group. Group 3. “Severe insulin resistance” group. Group 4. “Severe insulin resistance and beta-cell dysfunction” group. *aOR* adjusted odds ratio.

For the assessment of newly developed diabetic retinopathy, 2575 patients who underwent repeated fundus examinations annually were included. After a median follow-up of 5.8 years (min 180 days, max 6038 days), a significant increase in the risk was confirmed in the group 4 in the crude model (Supplementary Table 2). However, no significant difference was found in the risk of retinopathy occurrence between the groups after adjusting variable metabolic factors (Fig. 2a). For the assessment of newly developed CKD, 5685 patients who underwent repeated serum analysis were included. After a median follow-up of 4.6 years (min 61 days, max 5829 days), a significant increase in the risk was confirmed in the group 4 in the crude model (Supplementary Table 3). However, there was no significant difference in the risk of CKD development between the groups after adjusting variable metabolic factors (Fig. 2b). For the assessment of carotid atherosclerosis progression, 792 patients who underwent repeated carotid artery ultrasonography after 8–10 years of follow-up were included. The median follow-up duration was 9.2 years, and there was no significant difference between the groups (*p* = 0.843; Fig. 3).

To demonstrate the association between diabetes duration and change in subgroup distribution, we categorized the patients into quartiles of diabetes duration. As the T2DM duration increased, the proportion of patients in groups 1 and 4 decreased and increased, respectively (*p* for trend < 0.001; Supplementary Fig. 2).

## Discussion

**Figure 4:**
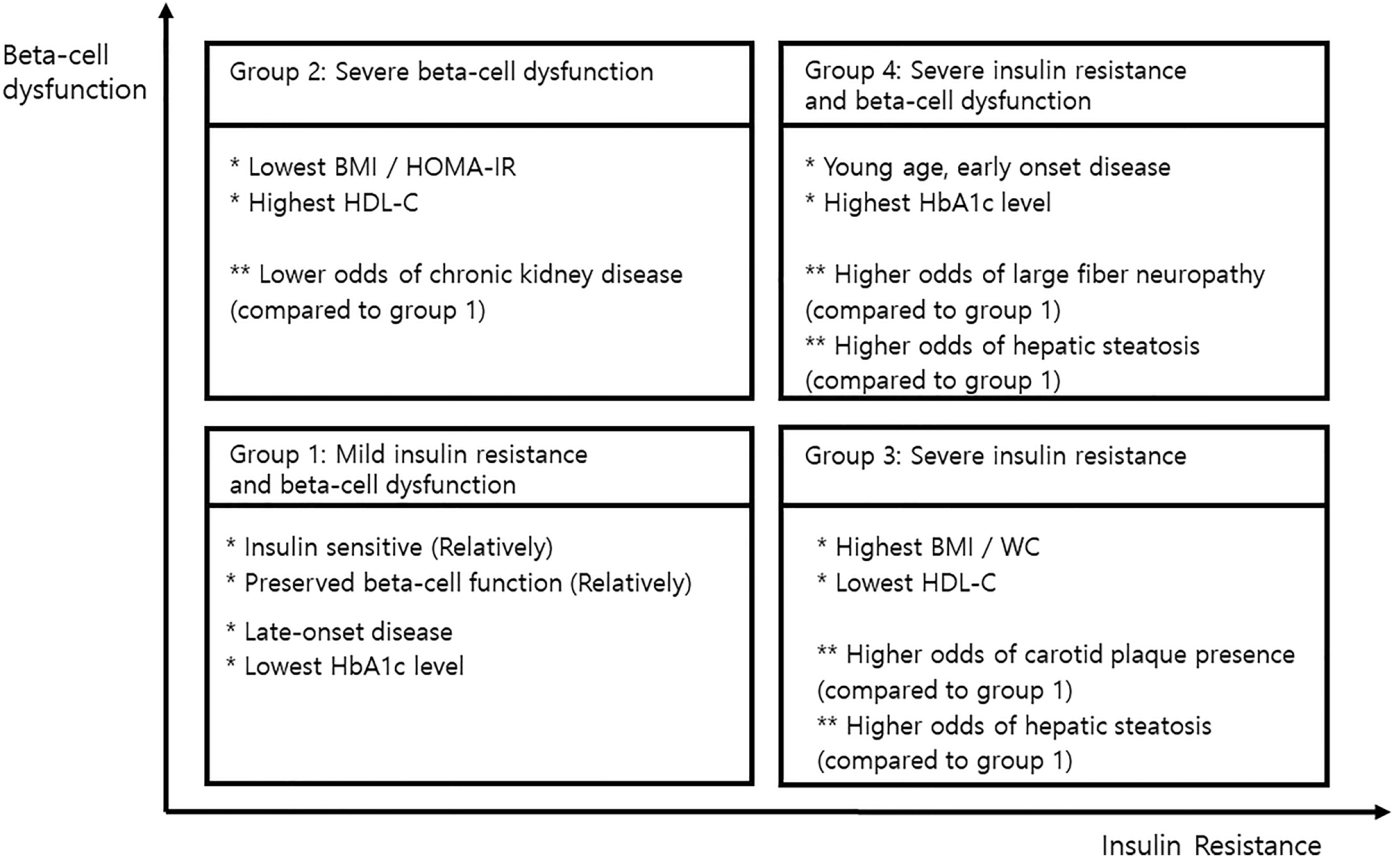
Summary of the group characteristics and risk of accompanying diabetic complications. *DM* diabetes mellitus, *BMI* body mass index, *WC* waist circumference, *HOMA* homeostatic model assessment, *IR* insulin resistance, *HDL* high-density lipoprotein.

A better understanding of different phenotypes of T2DM and how they relate to the complication risk is of great significance from the standpoint of administering personalized preventative and therapeutic interventions. In this study, patients with T2DM were classified into four groups according to insulin sensitivity and beta-cell function. These groups of patients had significantly different patient characteristics and odds of diabetic complications. However, there was no significant difference among the groups in retinopathy development, CKD progression, and plaque progression risk in the longitudinal follow-up analysis. The characteristics and diabetic complications in each group are summarized in Fig. 4. As T2DM duration increased, the proportion of patients in groups 1 and 4 decreased and increased, respectively.

Group 1 served as the reference group for the analysis. This group consisted of people whose insulin secretion function was relatively preserved and who were relatively insulin sensitive. This group had the shortest T2DM duration, highest age at T2DM diagnosis, lowest HbA1c level, and lowest odds of large fiber neuropathy. However, these characteristics did not lead to a low risk of long-term diabetic complications, including retinopathy, CKD, and carotid plaque progression. Group 2 included patients with low insulin secretion function but without high insulin resistance. These patients also had a long T2DM duration and the lowest BMI and WC. In a cohort study of patients with T2DM in Asia, Yeung et al.^17^ reported that patients with young-onset diabetes had higher mean concentrations of HbA1c and low-density lipoprotein cholesterol and a higher prevalence of retinopathy than patients with late-onset diabetes. Groups 2 and 4 had significantly higher HbA1c and LDL cholesterol levels than group 1 and showed characteristics that were similar to those of patient groups with young-onset diabetes. In the current study, group 2 showed the high incidence of diabetes retinopathy, but no significant increase in complication risk was confirmed after adjusting for variables, including HbA1c and diabetes duration. The high risk of complications in young-onset T2DM compared with late-onset T2DM was mainly due to a longer duration of hyperlyclemia^18^, and adjustment for HbA1c and duration of diabetes may attenuate the effect size for the risk of diabetic retinopathy. The accompanying odds of chronic kidney disease was lowest in group 2. This can be associated with the lowest insulin resistance value in group 2^19^. Compared with group 1, group 2 (severe beta-cell dysfunction) had no increased odds of diabetic complications.

Insulin secretion function was relatively preserved in group 3, but an increase in insulin resistance was evident. Group 3 had the highest BMI, WC and HOMA-IR. For patients in group 3, high insulin resistance may have played a main role in the development of diabetes rather than beta-cell dysfunction. The odds of hepatic steatosis and plaque presence were significantly higher. Insulin resistance and the progression of hepatic steatosis are closely related^20^. In the current study, the odds of accompanying hepatic steatosis was increased in groups with high insulin resistance (i.e., groups 3 and 4). Hepatic steatosis accompanied by insulin resistance was associated with an increased risk of carotid atherosclerosis^6^, and this association was also confirmed in group 3 of the current study. In group 4, insulin resistance was high, and insulin secretion function was low. This group had the lowest age at diabetes onset, the longest diabetes duration, and the highest HbA1c level. The highest odds of accompanying large fiber neuropathy and steatosis was also noted. In a previous study, diabetic neuropathy was shown to have a significant relationship with the quality of T2DM control and diabetes duration^21^.

When new developments of diabetic retinopathy and CKD were evaluated, a significant increase in the risk was confirmed in the group 4 in the crude model. This seems to be a result of reflecting the long T2DM duration and poor glycemic control, and it was no longer significant when various metabolic variables were adjusted. When new developments of carotid plaque were evaluated, no significant difference was found in the risk between the groups. Previous studies have shown that insulin resistance and glycemic control are independently and closely related to the risk of atherosclerosis progression^22,23^. Given that this study analyzed insulin resistance and insulin secretion function by categorizing the respective index values according to median values, the effect of these factors on the long-term risk of atherosclerosis was more likely to be diluted by other factors such as medication use, diet, and exercise. Similarly, in studies using the cluster technique, the unadjusted risk of coronary events and stroke differed between groups, but when adjusted for age or sex, the differences between groups tended to attenuated^3^. The results of the current study suggest that the T2DM subtype may reflect the current complication status but may not predict the future complication risk. When categorized according to T2DM duration, beta-cell dysfunction continued to progress as the T2DM duration increased, and the number of patients in groups 2 and 4 tended to increase. Therefore, the regular re-evaluation of insulin resistance and secretory function may help predict the current risk of diabetic complications.

A major limitation of this study is that it was based on a single-center cohort of Koreans with a relatively small number of participants. Considering that insulin resistance and insulin secretion capacity were defined on the basis of the median values within this group, the definition may change depending on the population. Patients with latent autoimmune diabetes in adults other than T2DM might not have been completely excluded in this study, as GADAs was tested only when type 1 diabetes or latent autoimmune diabetes in adults was strongly suspected. Selection bias could also be present because we only enrolled patients who underwent concurrent testing for fasting glucose, fasting insulin, and one or more complications. Lack of statistical power in longitudinal follow-up design analyzes may have affected the present non-significant results. The use of thiazolidinedione, and sulfonylurea, which are drugs that can significantly affect HOMA-B or HOMA-IR values, were corrected in the additional analysis. However, the use of other anti-diabetes medications may also affect the HOMA values.

Advantage of this study was that the evaluation of diabetic complication risk was conducted at the same clinic and was performed on the basis of records evaluated using the same criteria. This confirmed the cross-sectional association between HOMA-derived parameters and diabetic complications. In addition, the predictability of future complications using HOMA-derived parameters was analyzed through follow-up data analysis. Unlike with previous studies, this study was not limited to patients with new-onset T2DM. Therefore, the study can also be widely applied to patients with T2DM with a long duration of diabetes. The results of this study may contribute to guidance for a more focused evaluation of the risk of complications according to patient characteristics via a simple test for insulin resistance/insulin secretion function.

In conclusion, the pattern of complications varied according to the type of T2DM, which was simply classified on the basis of insulin resistance and beta-cell dysfunction. However, this model was not sufficient to predict the future risk of developing new diabetic complications.

## Supplementary Information

Supplementary Information.

## Data Availability

The datasets generated during and/or analysed during the current study are available from the corresponding author on reasonable request.
