# Supplementary material for: Differences in complication patterns in subgroups of type 2 diabetes according to insulin resistance and beta-cell function
Source: Sci Rep. 2022 Jun 7;12:9384. doi: 10.1038/s41598-022-13084-6 (PMC9174240; doi:10.1038/s41598-022-13084-6)
Supplement: Supplementary file 1 — Supplementary Information. [file 41598_2022_13084_MOESM1_ESM.docx]

Supplementary Figure 1. Odds of accompanying diabetic complications or hepatic steatosis by group according to insulin sensitivity and beta-cell function (without previous insulin users, additionally adjusted sulfonylurea use & thiazolidinedione use)


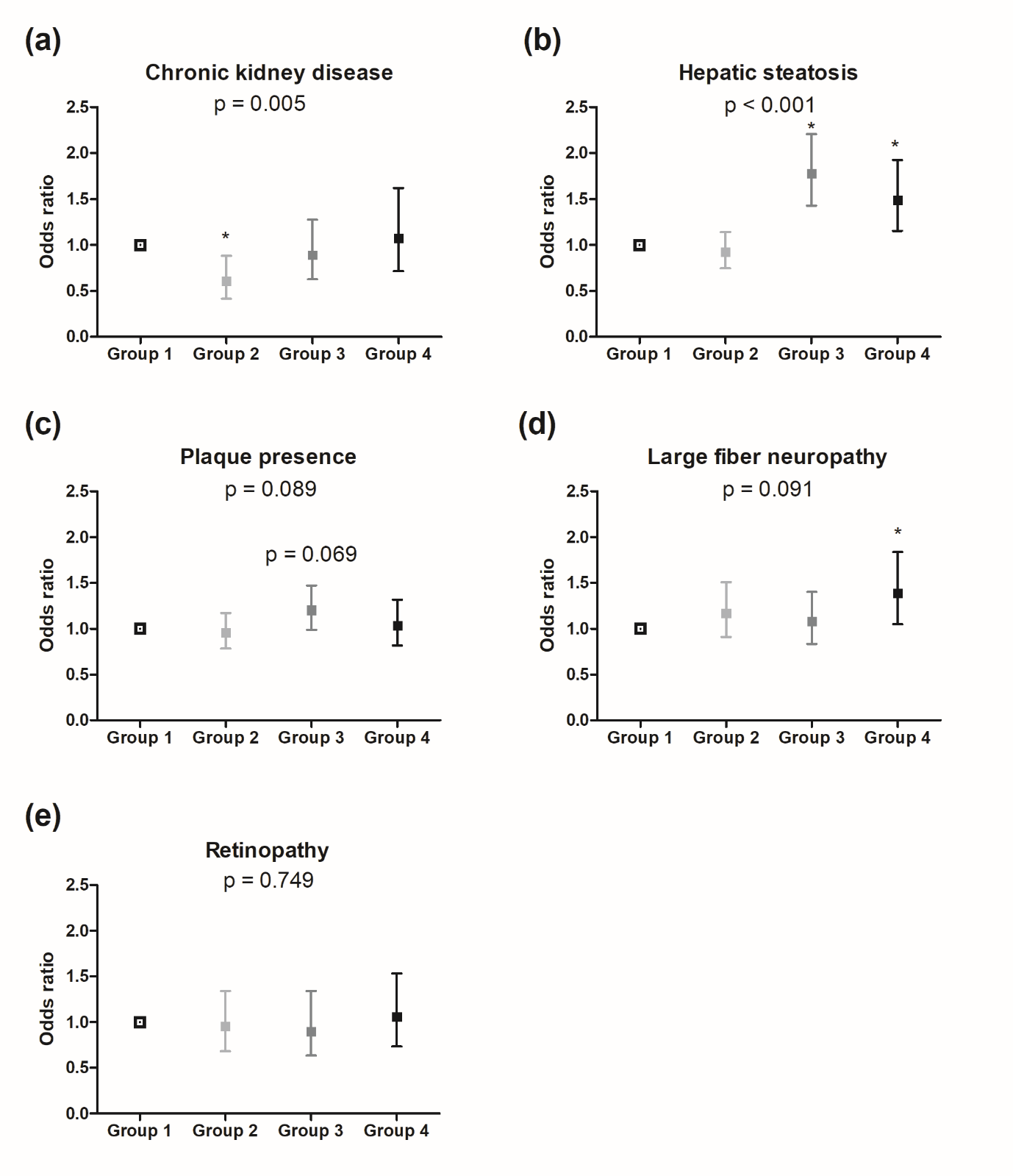


Odds ratios of (A) chronic kidney disease (eGFR < 60 mL/min/1.73 m^2^), (B) hepatic steatosis, (C) plaque formation, (D) diabetic neuropathy, and (E) diabetic retinopathy. The results were adjusted for age, sex, diabetes duration, systolic blood pressure, diastolic blood pressure, method of insulin measurement, time at enrollment, BMI, HbA1c levels, LDL cholesterol levels, eGFR (not included in the analysis depicted in Figure 1a), statin use, sulfonylurea use, thiazolidinedione use, smoking status, alcohol consumption, and physical activity.

**^*^**p values < 0.05, vs. group 1

Group 1. Reference (mild insulin resistance and beta-cell dysfunction) group.

Group 2. “Severe beta-cell dysfunction” group.

Group 3. “Severe insulin resistance” group.

Group 4. “Severe insulin resistance and beta-cell dysfunction” group.


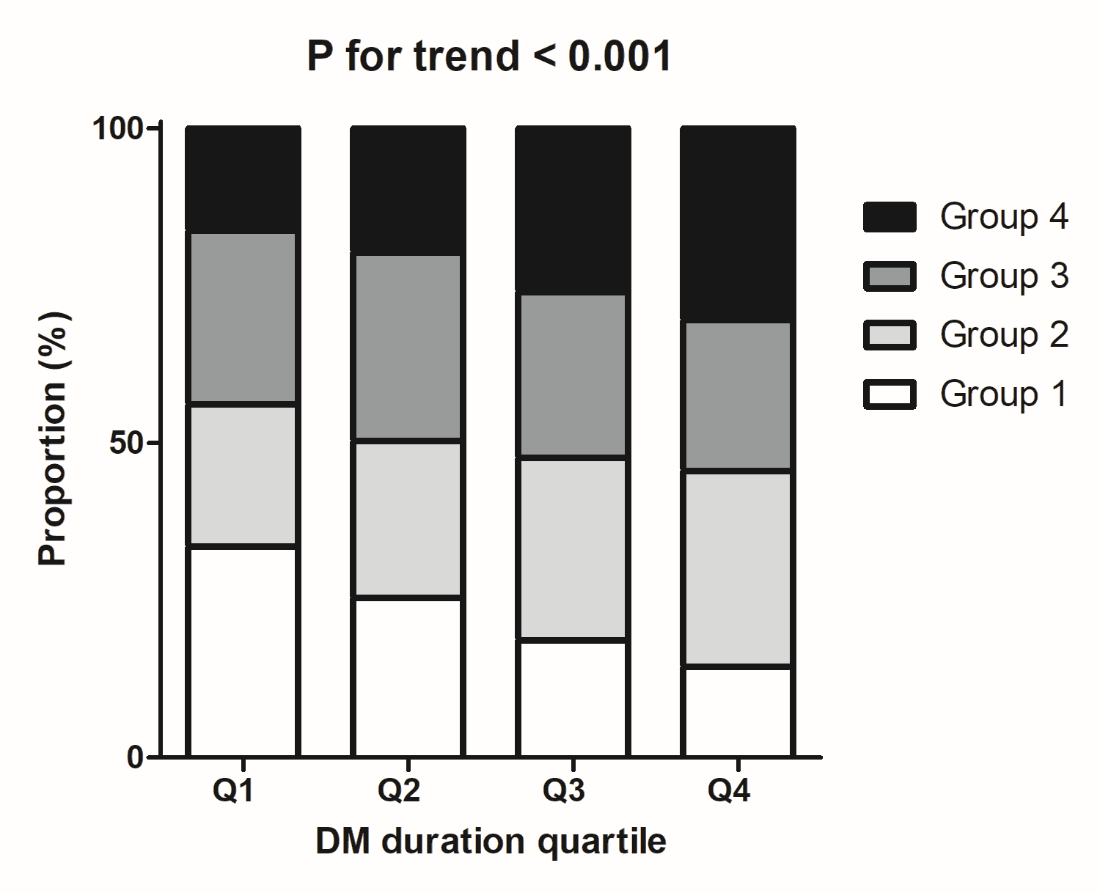


Supplementary Figure 2. Proportion of participants in each group according to the quartiles of diabetes duration

White, group 1; gray, group 2; dark gray, group 3; black, group 4

Q1, quartile 1 (diabetes duration < 2 years, n = 2,400); Q2, quartile 2 (diabetes duration of 2–5 years, n = 2,863); Q3, quartile 3 (diabetes duration of 6–10 years, n = 2,850); Q4, quartile 4 (diabetes duration > 10 years, n = 2,472)

Group 1. Reference (mild insulin resistance and beta-cell dysfunction) group.

Group 2. “Severe beta-cell dysfunction” group.

Group 3. “Severe insulin resistance” group.

Group 4. “Severe insulin resistance and beta-cell dysfunction” group.

Supplementary Table 1. Odds of accompanying diabetic complications or hepatic steatosis by group according to insulin sensitivity and beta-cell function

|  | Chronic kidney disease | p value | Heptaic steatosis | p value | Plaque presence | p value | Large fiber neuropathy | p value | Retinopathy | p value |
| --- | --- | --- | --- | --- | --- | --- | --- | --- | --- | --- |
| Group 1 | Reference | | Reference | | Reference | | Reference | | Reference | |
| Group 2 | **0.611**  **(0.420-0.889)** | **0.010** | 0.914  (0.739-1.130) | 0.408 | 0.961  (0.786-1.176) | 0.701 | 1.192  (0.925-1.535) | 0.174 | 0.971  (0.693-1.361) | 0.866 |
| Group 3 | 0.908  (0.637-.1.295) | 0.595 | **1.813**  **(1.459-2.252)** | **<0.001** | **1.238**  **(1.014-1.512)** | **0.036** | 1.087  (0.838-1.411) | 0.531 | 0.899  (0.634-1.276) | 0.553 |
| Group 4 | 1.085  (0.721-1.632) | 0.696 | **1.499**  **(1.161-1.935)** | **0.002** | 1.045  (0.823-1.328) | 0.718 | **1.397**  **(1.055-1.851)** | **0.020** | 1.06  (0.733-1.535) | 0.756 |

Odds ratios of (A) chronic kidney disease (eGFR < 60 mL/min/1.73 m^2^), (B) hepatic steatosis, (C) plaque formation, (D) diabetic neuropathy, and (E) diabetic retinopathy. The results were adjusted for age, sex, diabetes duration, hypertension, BMI, HbA1c levels, LDL cholesterol levels, eGFR (not included in the analysis depicted in Figure 1a), statin use, smoking status, alcohol consumption, and physical activity.

**^*^**p values < 0.05, vs. group 1

Group 1. Reference (mild insulin resistance and beta-cell dysfunction) group.

Group 2. “Severe beta-cell dysfunction” group.

Group 3. “Severe insulin resistance” group.

Group 4. “Severe insulin resistance and beta-cell dysfunction” group.

Supplementary Table 2. Risk of developing new retinopathy by group according to insulin sensitivity and beta-cell function

|  | Crude HR | 95% CI | p value |  | Adjusted HR | 95% CI | p value |
| --- | --- | --- | --- | --- | --- | --- | --- |
| Group 1 | 1 (Reference) | | |  | 1 (Reference) | | |
| Group 2 | 1.369 | (0.951-1.971) | 0.091 |  | 1.053 | (0.659-1.683) | 0.829 |
| Group 3 | 1.052 | (0.725-1.528) | 0.789 |  | 0.900 | (0.561-1.442) | 0.661 |
| Group 4 | 1.867 | (1.314-2.653) | <0.001 |  | 1.27 | (0.766-2.106) | 0.355 |

The results were adjusted for age, sex, diabetes duration, systolic blood pressure, diastolic blood pressure, time of enrollment, BMI, HbA1c levels, LDL cholesterol levels, eGFR, statin use, smoking status, alcohol consumption, and physical activity.

Group 1. Reference (mild insulin resistance and beta-cell dysfunction) group.

Group 2. “Severe beta-cell dysfunction” group.

Group 3. “Severe insulin resistance” group.

Group 4. “Severe insulin resistance and beta-cell dysfunction” group.

Supplementary Table 3. Risk of developing new chronic kidney disease by group according to insulin sensitivity and beta-cell function

|  | Crude HR | 95% CI | p value |  | Adjusted HR | 95% CI | p value |
| --- | --- | --- | --- | --- | --- | --- | --- |
| Group 1 | 1 (Reference) | | |  | 1 (Reference) | | |
| Group 2 | 1.084 | (0.831-1.413) | 0.552 |  | 0.723 | (0.514-1.018) | 0.063 |
| Group 3 | 1.236 | (0.962-1.589) | 0.098 |  | 1.059 | (0.771-1.455) | 0.724 |
| Group 4 | 1.421 | (1.095-1.843) | 0.008 |  | 0.836 | (0.570-1.227) | 0.360 |

The results were adjusted for age, sex, diabetes duration, systolic blood pressure, diastolic blood pressure, time of enrollment, BMI, HbA1c levels, LDL cholesterol levels, statin use, smoking status, alcohol consumption, and physical activity.

Group 1. Reference (mild insulin resistance and beta-cell dysfunction) group.

Group 2. “Severe beta-cell dysfunction” group.

Group 3. “Severe insulin resistance” group.

Group 4. “Severe insulin resistance and beta-cell dysfunction” group.
